# Human Gut Bacteria Are Sensitive to Melatonin and Express Endogenous Circadian Rhythmicity

**DOI:** 10.1371/journal.pone.0146643

**Published:** 2016-01-11

**Authors:** Jiffin K. Paulose, John M. Wright, Akruti G Patel, Vincent M. Cassone

**Affiliations:** Department of Biology, University of Kentucky, Lexington, KY, 40506, United States of America; University of Texas Southwestern Medical Center, UNITED STATES

## Abstract

Circadian rhythms are fundamental properties of most eukaryotes, but evidence of biological clocks that drive these rhythms in prokaryotes has been restricted to Cyanobacteria. In vertebrates, the gastrointestinal system expresses circadian patterns of gene expression, motility and secretion *in vivo* and *in vitro*, and recent studies suggest that the enteric microbiome is regulated by the host’s circadian clock. However, it is not clear how the host’s clock regulates the microbiome. Here, we demonstrate at least one species of commensal bacterium from the human gastrointestinal system, *Enterobacter aerogenes*, is sensitive to the neurohormone melatonin, which is secreted into the gastrointestinal lumen, and expresses circadian patterns of swarming and motility. Melatonin specifically increases the magnitude of swarming in cultures of *E*. *aerogenes*, but not in *Escherichia coli* or *Klebsiella pneumoniae*. The swarming appears to occur daily, and transformation of *E*. *aerogenes* with a flagellar motor-protein driven lux plasmid confirms a temperature-compensated circadian rhythm of luciferase activity, which is synchronized in the presence of melatonin. Altogether, these data demonstrate a circadian clock in a non-cyanobacterial prokaryote and suggest the human circadian system may regulate its microbiome through the entrainment of bacterial clocks.

## Introduction

In contrast to the situation in the animal clock, which involves a transcriptional, translational feedback of “clock genes” such as *Per*, *Cry*, *Bmal1* and *Clock*, prokaryotic circadian clocks, demonstrated only in the cyanobacterium *Synechococcus elongatus*, are post-transcriptional in nature. These bacteria express circadian patterns of gene expression, photosynthesis and nitrogen fixation [1–3], but the molecular mechanism for this cyanobacterial clock is the result of rhythmic autokinase activity of the hexamer-forming ATPase KaiC that is enhanced by KaiA binding and subsequent autophosphatase activity of KaiC that is modulated by KaiB binding to the KaiA-KaiC complex [4]. Remarkably, the three purified proteins, when provided free ATP, exhibit rhythmic phosphorylation of KaiC *in vitro* for many cycles [5]. Although *S*. *elongatus* is the only cyanobacterium studied in much detail, the Kai proteins are found extensively within the Phylum Cyanobacteria [6]

As stated above, vertebrate circadian organization results from the rhythmic expression of “clock genes” whose products interact in a dynamic transcription/translation feedback loop [1,7] “Positive elements” *Clock* and *Bmal1* are transcribed and translated in the cytoplasm, where they dimerize, reenter the nucleus and activate expression of genes containing an E-box element in their promoter regions. Among these, “negative elements” *Period* (*per1*, *2* and *3*) and *Cryptochrome* (*cry1* and *2*) are transcribed, translated and then feedback within the nucleus by interfering with *Clock/Bmal1* transcriptional activation [1,7,8] The molecular feedback loop is expressed in multiple tissues in the body, where they regulate rhythmic processes locally, but they are coordinated by pacemakers such as the hypothalamic suprachiasmatic nucleus (SCN) in mammals [9].

Among the vertebrate peripheral tissues that express circadian rhythms is the gastrointestinal system, which exhibit circadian rhythms in gene expression (including clock genes), motility and secretion *in vivo* and *in vitro* [10–12]. These rhythms depend upon a patent molecular clock, since they are abolished in *per1/per2* double knockout mice [12]. They are also coordinated by SCN input via the sympathetic nervous system [13].

The emerging role of the gut microbiome as an important modulator of gastrointestinal function has recently included the role of circadian rhythms. Recent studies have suggested that microbial signaling plays a critical role in homeostatic maintenance of intestinal function along with the host circadian mechanism [14,15]. Further studies have expanded this view and have shown that disruption of the circadian clock, either via dietary restriction or phase shifting (e.g. jet-lag) affects temporal distribution of the gut microbiome constituents [16–19]. While it is clear from these studies that commensal bacteria and gut tissues do communicate, it is not clear which signal or signals the microbiome exploits to sustain its own homeostasis.

Here we present evidence for one possible signal, the indole hormone melatonin, which is present at high levels in the gut and which induces swarming activity in a clinical isolate of *Enterobacter aerogenes*. Further investigation of the motility patterns in this bacterium evinced an endogenous circadian rhythm within cultures, which is enhanced and synchronized by melatonin.

## Materials and Methods

Strains and culture conditions: *E*. *aerogenes* and *E*. *coli* clinical isolates (gift from Dr. John Seabolt, U. of Kentucky), *K*. *pneumoniae* Isolate-1 (NR-15410, BEI resources, NIAID, NIH), and DH5α with *luxcdabe* driven by the promoter region of *MotA* [20] (gift from Brian Ahmer, Ohio State University), were initially cultured in LB Broth at 37°C in a shaking incubator. Motility assays were conducted on Eosin-Methylene Blue Agar (EMB) plates [21] with a 50% reduction in agar to facilitate motility. All chemicals used in motility assays were purchased from Sigma (St. Louis, MO) and diluted in water.

Motility Assays: 100mm petri dishes were visually divided into quadrants, filled with 30mls of EMB agar with or without specified concentration of chemicals, and allowed to dry for ~4 hours in a sterile hood. 2μl of overnight culture were stabbed and released into the center of each quadrant and allowed to grow for 48 hours at 37°C. Each plate was imaged on a light box by digital camera using qCapture Pro software (Media Cybernetics, MD) and areas measured by ImageJ [22].

Transformation of MotA::luxcdabe into E. aerogenes: *E*. *aerogenes* were made competent by CaCl_2_ method and *MotA*::*luxcdabe* plasmid extracted from the host strain was transformed into *E*. *aerogenes* by heat shock. Transformants were selected for on tetracycline-supplemented medium and stored as glycerol stocks for future studies.

Bioluminescence monitoring: 2μl of overnight cultures were stabbed and released into the center of 35mm culture dishes containing 5mls of EMB agar with or without 1nM melatonin. Plates were sealed with 40mm cover glass by sterile vacuum grease and placed into an automated photomultiplier-based bioluminescence recorder (Lumicycle, Actimetrics, Il). Each sample was counted for 70 seconds on a rotating platform. Raw bioluminescence baselines were subtracted using a 24-hour running average via Lumicycle Analysis software (Actimetrics, IL). Cultures were photographed as above and used for illustrative purposes here.

Bioinformatics: Initial protein searches were performed by BLASTp (NCBI) using human MEL1A and MEL1B protein sequences against a protein database from the curated human microbiome project (HMP) repository (NCBI). Clock protein comparisons were performed by PsiBLAST program (NCBI) using Uniref_50 clusters against the available proteomes of *E*. *aerogenes* strains KCTC2190 and EA1509E (Uniprot.org taxonomy IDs 1028307 and 935296, respectively). Unique microbial proteins were aligned to the original clock gene clusters using MUSCLE and trees generated by PhyML software with 100 bootstraps.

Motif analysis: KAI complex protein sequences, including positive PsiBLAST hits above, were entered into the online MEME suite ([23] http://meme.nbcr.net/meme) under Multiple Em for Motif Elicitation (MEME) and subsequent Motif Alignment and Search Tool (MAST, [24]) The output of MAST for each protein is included here in supplemental data.

Statistics: Circadian rhythmicity was determined by Circwave, Circwave Batch software v3.3 [25] and periodogram analysis [26]. Each day of bioluminescence recording was separated and analyzed for periods of 24 hours (Circwave and periods between 19 and 28 hours (Circwave Batch, p<0.02). Periodogram analysis was performed using R statistical program and the GeneCycle package [26] followed by Fisher's exact g Test to obtain p-values of each culture. Spread/motility measures, periods, amplitudes, and damping coefficients were compared by 1- or 2-way ANOVA, where appropriate. All analyses were performed using SigmaStat software (Systat, CA).

## Results and Discussion

We hypothesized that one potential human signal that may affect gastrointestinal microbiota is the secretion of melatonin into the lumen of the gut. Although melatonin is widely regarded as a pineal and retinal neuromodulator of circadian and photoperiodic function [27, 28], it is present throughout the gastrointestinal system [27–29], in part from pineal melatonin secretion [30, 31], but there is evidence for melatonin biosynthetic enzymes in biliary cholangiocytes, enterochromaffin cells and intestinal mucosa [31, 32]. In addition, many foods contain melatonin [27, 31]. In all, melatonin content has been reported to be 10-400x levels found in the serum [31, 32]. We identified from metagenomics data in GenBank several enteric bacteria that expressed sequences with 24–42% identity to known melatonin binding sites in the human genome (S1 Fig). These included receptors in *Enterobacter aerogenes*, but not in *Escherichia coli* or *Klebsiella pneumoniae*.

**Fig 1:**
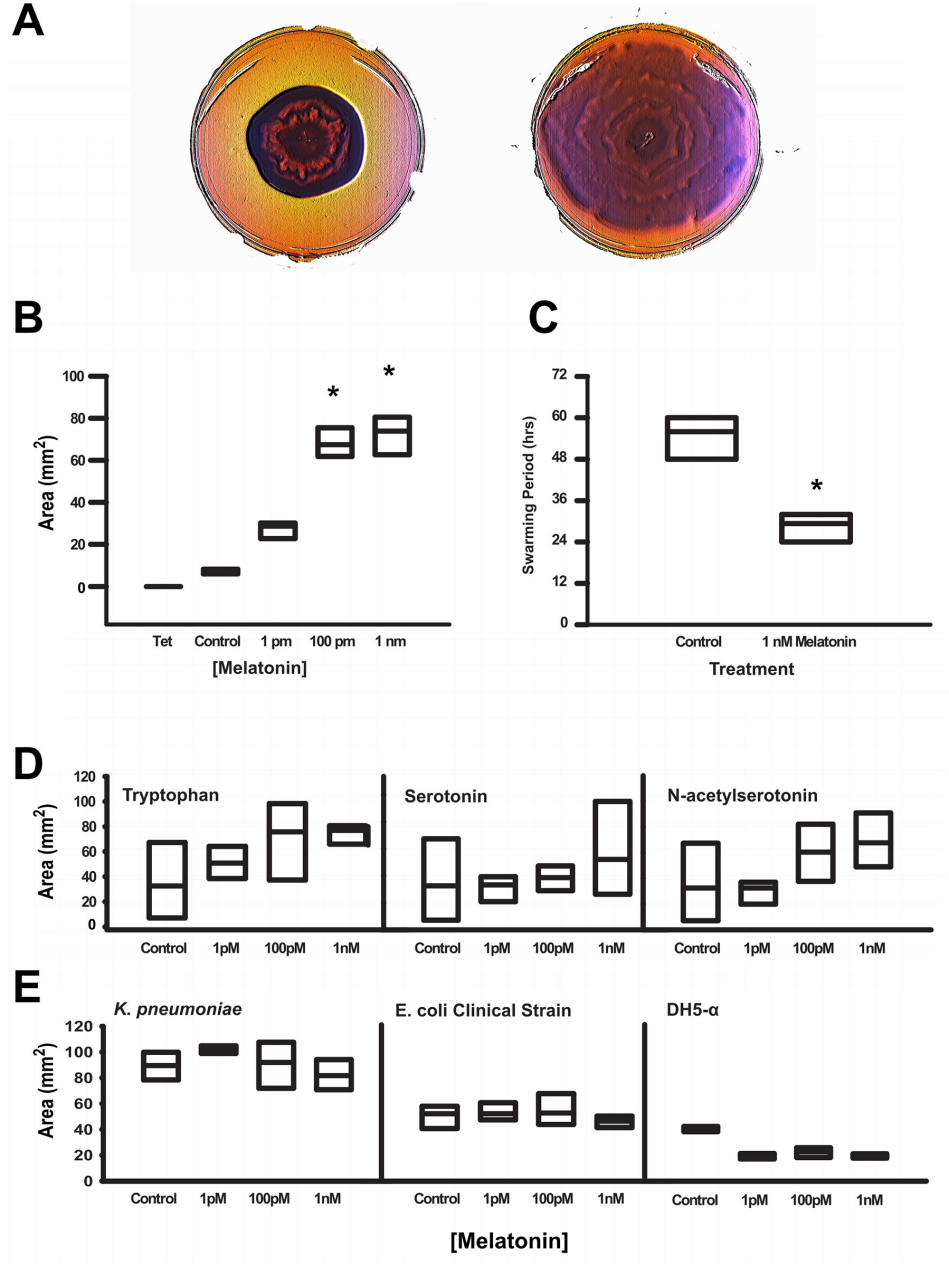
Swarming behavior in *E*. *aerogenes* is induced by melatonin and occurs with a circadian frequency. A Swarming behavior in control treated (left cultures vs. treatment with 1nM melatonin (right. Images were equally enhanced using “Bump Map” in GIMP software to highlight banding patterns. B The increase in swarming was only seen at 100pM and 1nM concentrations of melatonin, * = p value < 0.001 compared to vehicle treated cultures, n = 16 cultures per treatment. C Period of swarming as calculated by the number of rings observed per culture period of 4 days in n = 16 cultures per treatment, * = p value < 0.001. D Area of bacterial spread was unaffected by tryptophan (left, serotonin (middle and N-acetylserotonin (right, n = 16 cultures per treatment. E Melatonin did not affect growth in *K*. *pneumoniae* (left or clinical or lab strains of *E*. *coli* (middle and right, respectively, n = 16 cultures per strain per treatment.

**Fig 2:**
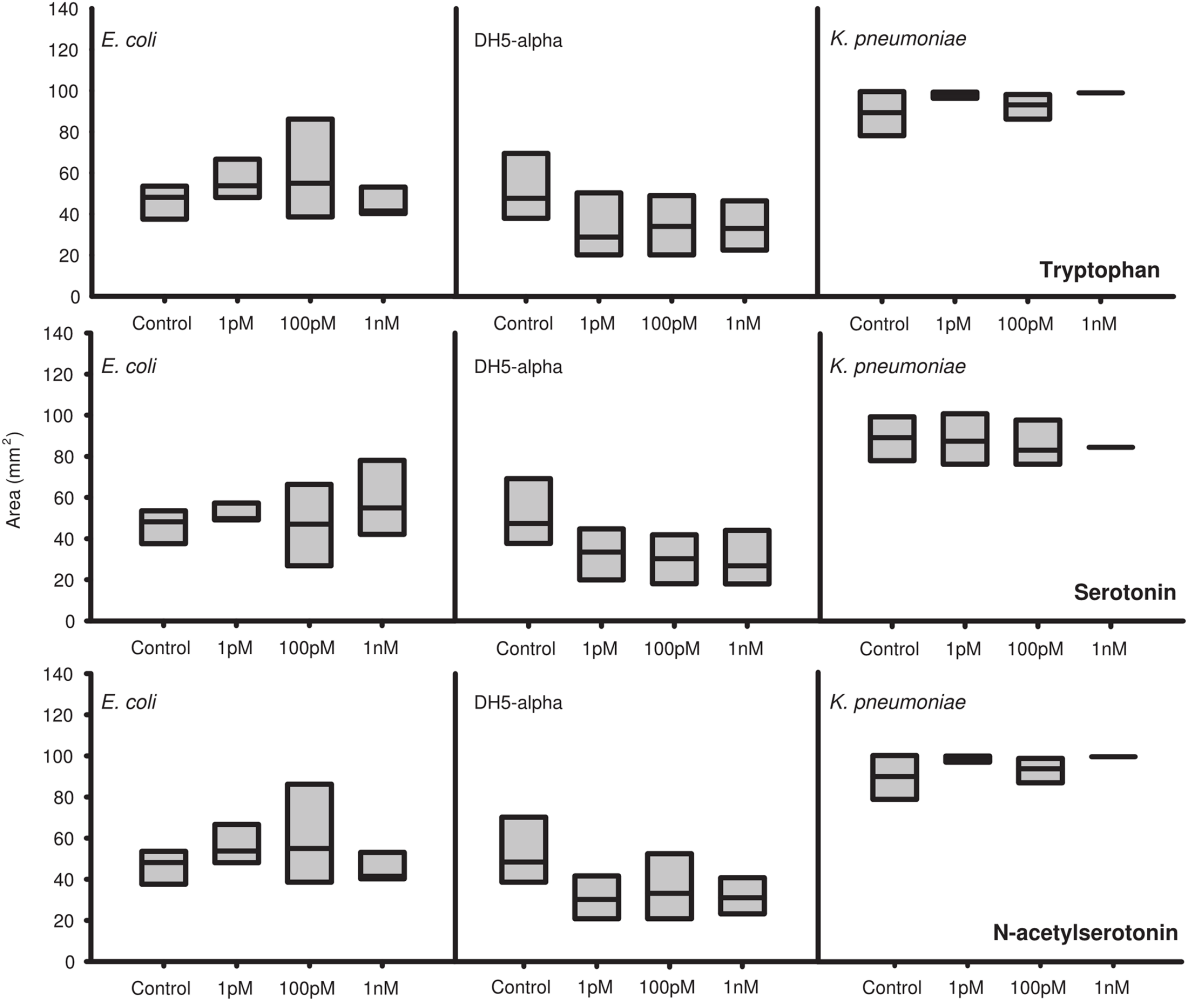
Neither lab nor clinical strains of *E*. *coli* nor *K*. *pneumoniae* show swarming response to other indoles. Cultures of clinical isolates of *E*. *coli* (left panels, DH5-α (middle panels, and *K*. *pneumoniae* (right panels were tested for swarming/growth in the presence of tryptophan (top row, serotonin (middle row, and N-acetylserotonin (bottom row, n = 16 cultures per strain per treatment.

Colonies formed by clinical isolates of *Enterobacter aerogenes*, a Gram negative, indole-negative motile bacterium, proliferated on semi-solid Agar significantly more rapidly in the presence of melatonin in a specific, dose-dependent fashion, with maximal response coinciding within the physiological range of gut melatonin levels (Fig 1A and 1B and S2 Fig). This effect was specific for melatonin, as *E*. *aerogenes* spread further in the presence of melatonin than in the presence of equimolar concentrations of tryptophan, serotonin or N-acetylserotonin (Fig 1D). In contrast, *Klebsiella pneumoniae*, a closely related but non-motile, indole-negative member, and *Escherichia coli*, an indole-positive but motile member of the Enterobacteriacea Family, do not respond to melatonin or the other indoles tested (Figs 1E and 2).

The larger cultures of *E*. *aerogenes* in the presence of melatonin exhibited patterns of swarming within the cultures, evidenced by stereotypical, concentric rings of colonies (Fig 1A), similar to recently reported diurnal swarming in *Listeria monocytogenes* [33] and identical to the bulls-eye pattern of swarming commonly seen in *Proteus mirabilis*, another intestinal commensal that is also in the Enterobacter family [34]. These patterns were less apparent in the smaller, control cultures of *E*. *aerogenes* in melatonin’s absence (S2 Fig). Remarkably, the number of rings consistently coincided with the number of incubation days. Calculation of banding periodicity—the number of bands visually observed divided by the number of hours of incubation—revealed a period of much greater than 24 hours in control-treated cultures. In contrast, in 1nM melatonin’s presence, the period of swarming behavior was 25.1 ± 1.4 (S.D. hours (Fig 1C).

**Fig 3:**
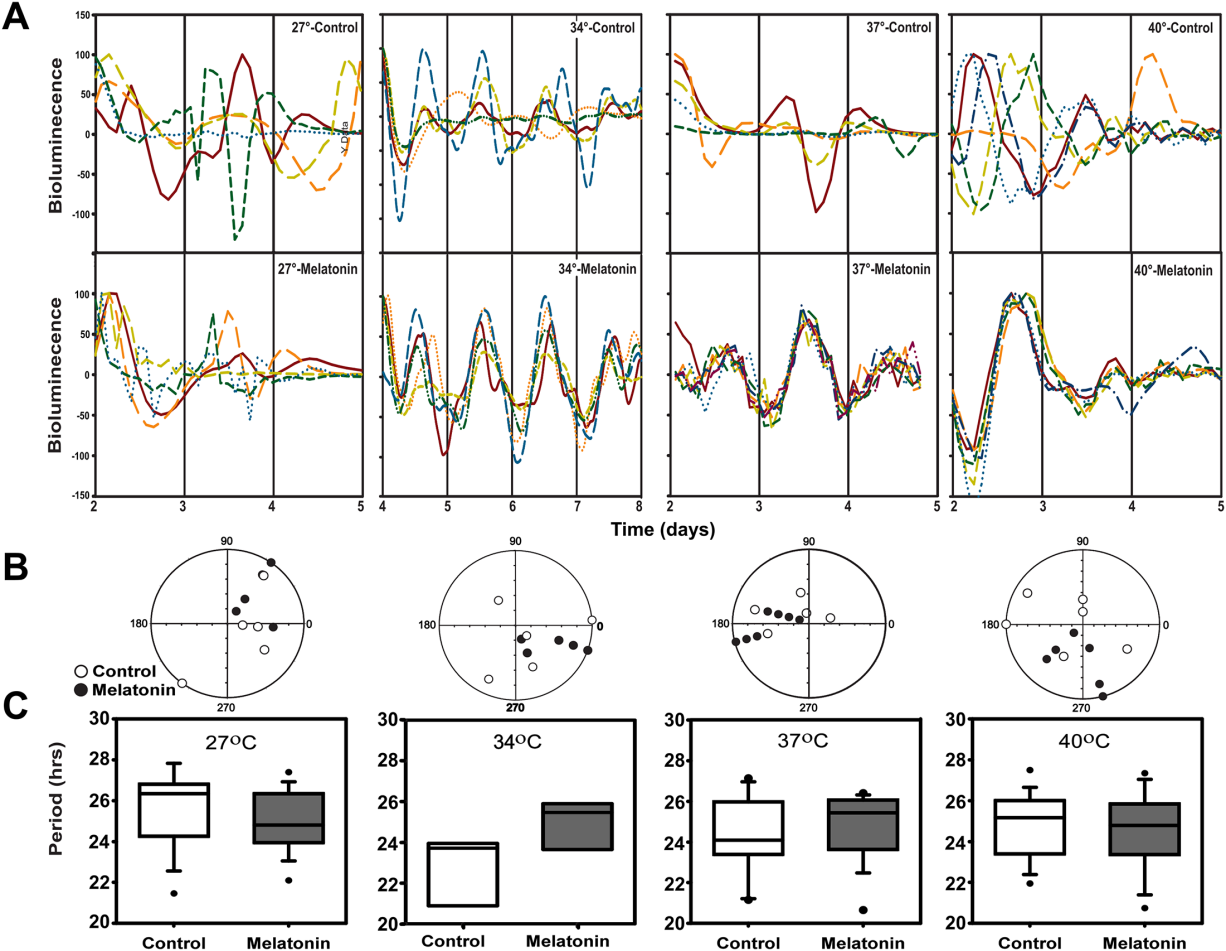
Bioluminescence recording of *MotA*::*luxcdabe* transformed *E*. *aerogenes* confirms a temperature compensated circadian rhythm. A) Normalized bioluminescence rhythms from control-treated (top panels) and melatonin-treated (bottom panels) cultures show circadian rhythms at (from left to right) 27° (n = 5/treatment), 34° (n = 5/treatment), 37° (n = 5 control and 7 melatonin-treated) and 40° (n = 6 control and 6 melatonin-treated). Time scales represent days after plates were inoculated with bacteria, which varied in the amount of time needed to stabilize and begin outgrowth. B) Periodogram analysis-derived peak phases of rhythmic cultures from (A) reveal that control-treated cultures (white circles) show greater variation in peak phase than melatonin-treated cultures (black circles), which are more synchronized at all three temperatures. C) Periods of rhythms varied between 22 and 28 hours among temperature and melatonin treatments, but were not significantly affected by temperature or melatonin.

From the above banding period data, we hypothesized that the swarming rhythms might represent the output of a circadian clock. To test this, cultures of *E*. *aerogenes* were transformed to express luciferase using a *luxcdabe* construct driven by the MotA promoter [20] (S3A Fig). Bioluminescence from these cultures measured in a Lumicycle photomultiplier system indicated robust circadian patterns in 31–44% of cultures when maintained in temperatures ranging from ambient 27°C to those corresponding to human body temperatures (T_B_) of 34°C, 37°C and 40°C (Fig 3A–3C). The circadian periods of these bioluminescence rhythms were temperature compensated with a Q_10_ = 0.96 from 27°C to 40°C. While there was no effect of melatonin on circadian period (Fig 3C), there was a significant effect of melatonin on the phase of peak bioluminescence (Fig 3B). In the absence of melatonin, the circadian phases of multiple replicates were highly variable. However, in the presence of 1 nM melatonin the phases of these rhythms were synchronized, especially at temperatures closely corresponding to body temperature (34–37°C) (Fig 3A and 3B). In contrast, the plasmid donor strain of DH5-α *E*. *coli* failed to exhibit daily patterns of bioluminescence in the presence or absence of melatonin, despite having a 5-fold higher raw bioluminescence level (S3B and S3C Fig), which may be attributed to a higher plasmid copy number.

This is the first demonstration of a circadian clock in a prokaryote outside Phylum Cyanobacteria. The fact that this species exists primarily as a commensal bacterium raises the possibility that the circadian clockworks driving these rhythms in *E*. *aerogenes* may have arisen from horizontal gene transfer of human and/or ancestral vertebrate clock genes into these bacteria [35, 36]. However, comparison of the *E*. *aerogenes* proteome to known members of the vertebrate biological clock mechanism revealed no relationship between BMAL1, CLOCK, OR PER1 and any sequence within the *E*. *aerogenes* proteome (S4A, S4B and S4C Fig, respectively).

On the other hand, comparison of the *E*. *aerogenes* proteome data set to the cyanobacteria *KAIABC* complex revealed several sequences nested within trees for each of the *Kai* proteins (S4D, S4E and S4F Fig). Although position-specific iterated BLAST (PsiBLAST) provided significant alignments, motif-specific analysis using MEME and MAST software showed little similarity to conserved motifs within the KAI proteins (S5 Fig). Despite a lack of similarity at the sequence level, there may be an underlying similarity in cellular functions of related proteins. One KaiC ortholog found here, Dephospho-Coa Kinase, is also known to act with a phosphatase in bacteria and mammals, with the latter relationship in the form of a bi-functional single enzyme [37–39]. In *S*. *elongatus*, the Kai complex drives circadian rhythms of multiple processes through a post-translational molecular mechanism that persists even outside the bacterial cell; combination of the three Kai proteins and ATP reconstitutes a circadian pattern of phosphorylation and dephosphorylation for many cycles *in vitro* [5]. The major component of the complex, KaiC, expresses both kinase and phosphatase activities, the latter of which occurs in a manner similar to ATP synthase [40]. *In vivo*, this oscillator responds to light, temperature, and metabolic state thorugh the *CikA*, *LdpA*, and *Pex* pathways, each of which can entrain the Kai oscillator to environmental cues [2, 3, 41]. This relatively simple oscillator in turn regulates a wide array of processes through transcriptional regulation [42, 43]. Other factors must influence this oscillator, however, since *in vivo*, the periods of multiple circadian rhythms differ, depending on the promoter, the presence or absence of promoter recognition subunits of RNA polymerase, and environmental conditions, including light intensity and growth phase of the culture [44]. Our data cannot exclude this possibility in *E*. *aerogenes*, as we have only examined rhythmicity as it manifests in MotA motor protein expression, which—although an established proxy for motility—is likely to be an output of the mechanism. Alternatively, but not exclusively, circadian rhythms in *E*. *aerogenes* may derive from rhythmic peroxiredoxin activity, since this mechanism has been identified only recently in eukaryotes as well as prokaryotes [45]. Bioinformatics analysis reveals several sequences that share similarity to peroxiredoxin and thiol redoxins from various taxa (S5G Fig). Contrary to earlier studies investigating the structure and function of KaiB and SasA proteins [46, 47] our analysis showed no similarity between KaiB and thioredoxins or between KaiB orthologs and peroxiredoxin orthologs. However, the candidate proteins from our analysis are all linked to redox-sensitive pathways, including the manganese transporter MntH that initiated this investigation [48]. Recent reports of the anti-oxidant properties of melatonin in a neurodegenerative mouse model would support a mechanism involving melatonin and redox-state sensing [49]. We are currently exploring these candidate proteins to determine the mechanism or mechanisms behind the observed rhythms.

**Fig 4:**
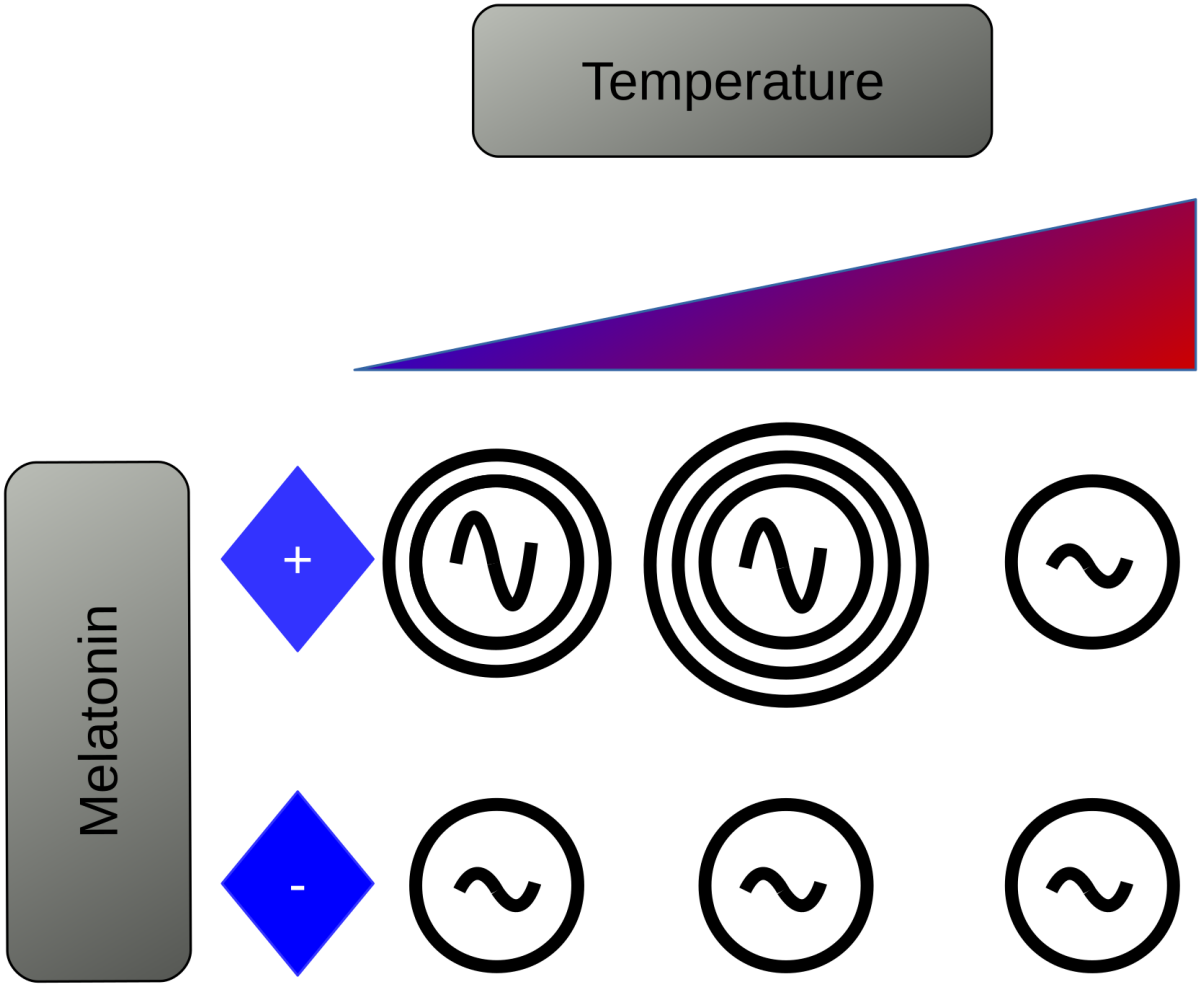
The data presented here show that a clinical isolate of *E*. *aerogenes* expresses a circadian rhythm in MotA expression and displays a swarming response to melatonin in a dose- and temperature-dependent manner.

Importantly, the present observations indicate that at least one member of the human microbiome may synchronize to its host through synchronization of an endogenous, temperature-compensated circadian clock. The detailed mechanism for this synchronization is at this stage not completely known. However, it is remarkable that the presence of melatonin in the culture medium synchronizes the periodicity and phases of multiple clonal populations across different culture plates (Fig 4). The latter phenomenon suggests melatonin as a novel source of host-commensal communication within the gut, if not the internal Zeitgeber itself. The existence of a circadian rhythm within a commensal bacterium that responds to an endocrine signal that is regulated by the circadian mechanism of the host gives further credence to the concept of the microbiome as a “meta-organism”; one with an endogenous clock that is entrained by its host's clock-driven signals. If we regard our own circadian mechanism as an evolved adaptation to environmental phenomena governed by 24-hour periods, organs and organ systems could be perceived as the entraining environment for resident microflora. As such, perturbations to the environment (i.e. circadian disruptions) will affect rhythms within the microbiome as previously demonstrated [17,19]. However, it is not known whether or not, nor to what extent, the microbiome can recover from these challenges. Furthermore, the effect of host: commensal signaling is likely not relegated to one species, as we are limited to here, but to the community at large. If this phenomenon modulates quorum sensing, as is suggested by observations, there would be systemic alterations to the microbial community as a whole, as well as to the physiology of the host.

## Supporting Information

S1 Fig Multiple sequence alignment of protein BLAST hits to human MEL1A and MEL1B receptors show several areas of identity with protein sequences taken from the Human Microbiome Project (HMP). Alignments shown are a selection of positive BLAST hits (e-value < 0.001) aligned using MUSCLE that show several conserved residues and regions of high identity.
(TIF)

S2 Fig Exposure to physiological levels of melatonin induce swarming in *E*. *aerogenes*. 100mm EMB agar plates were inoculated with 2ul of overnight cultures (n = 4/plate, replicated with 4 different starter cultures) and incubated for 48 hours. Rosette patterns of swarming increased with increasing concentrations of melatonin on the plates.
(TIF)

S3 Fig *MotA*::*luxcdabe* is expressed rhythmically in *E*. *aerogenes*, not in DH5-α. A Representative map of plasmid pRG19 showing MotA upstream of *luxcdabe* complex and tetracycline resistance. B) DH5-α cultures (left) are not rhythmic regardless of presence of melatonin, however, raw trace of *E*. *aerogenes* cultures (right) transformed with *MotA*::*luxcdabe* plasmid show rhythmic expression with damping over time both in the presence and absence of melatonin. C) Melatonin increased the average amplitude of cultures exhibiting circadian rhythms at 27°C and 37°C, but not 40°C, * = p value < 0.05 as tested by one-way ANOVA. D) Neither temperature nor melatonin affected the damping rate of the cultures exhibiting circadian rhythms.
(TIF)

S4 Fig Phylogenetic relationships exist between Cyanobacteria clock proteins and *E*. *aerogenes*, not vertebrate clock proteins. Bootstrapped trees (iterations shown between branches) show no homology among *E*. *aerogenes* proteins and vertebrate clock proteins BMAL1 (A), CLOCK (B), or PER1 (C). Similar analyses using Uniprot clusters of KAI A (D), KAI B (E), and KAI C (F) show potential homology with specific *E*. *aerogenes* proteins. G) *E*. *aerogenes* proteins share conservation with redox-related proteins across several taxa.
(TIF)

S5 Fig Kai protein orthologs in *E*. *aerogenes* do not share motif-level similarity with other Kai proteins. Proteins with sequence homology via PSI-BLAST share some motif-level sequences with A) KaiA and B) KaiB, but not C) KaiC.
(TIF)
