# Supplementary figures and images for: Human Gut Bacteria Are Sensitive to Melatonin and Express Endogenous Circadian Rhythmicity
Source: PLoS One. 2016 Jan 11;11(1):e0146643. doi: 10.1371/journal.pone.0146643 (PMC4709092; doi:10.1371/journal.pone.0146643)

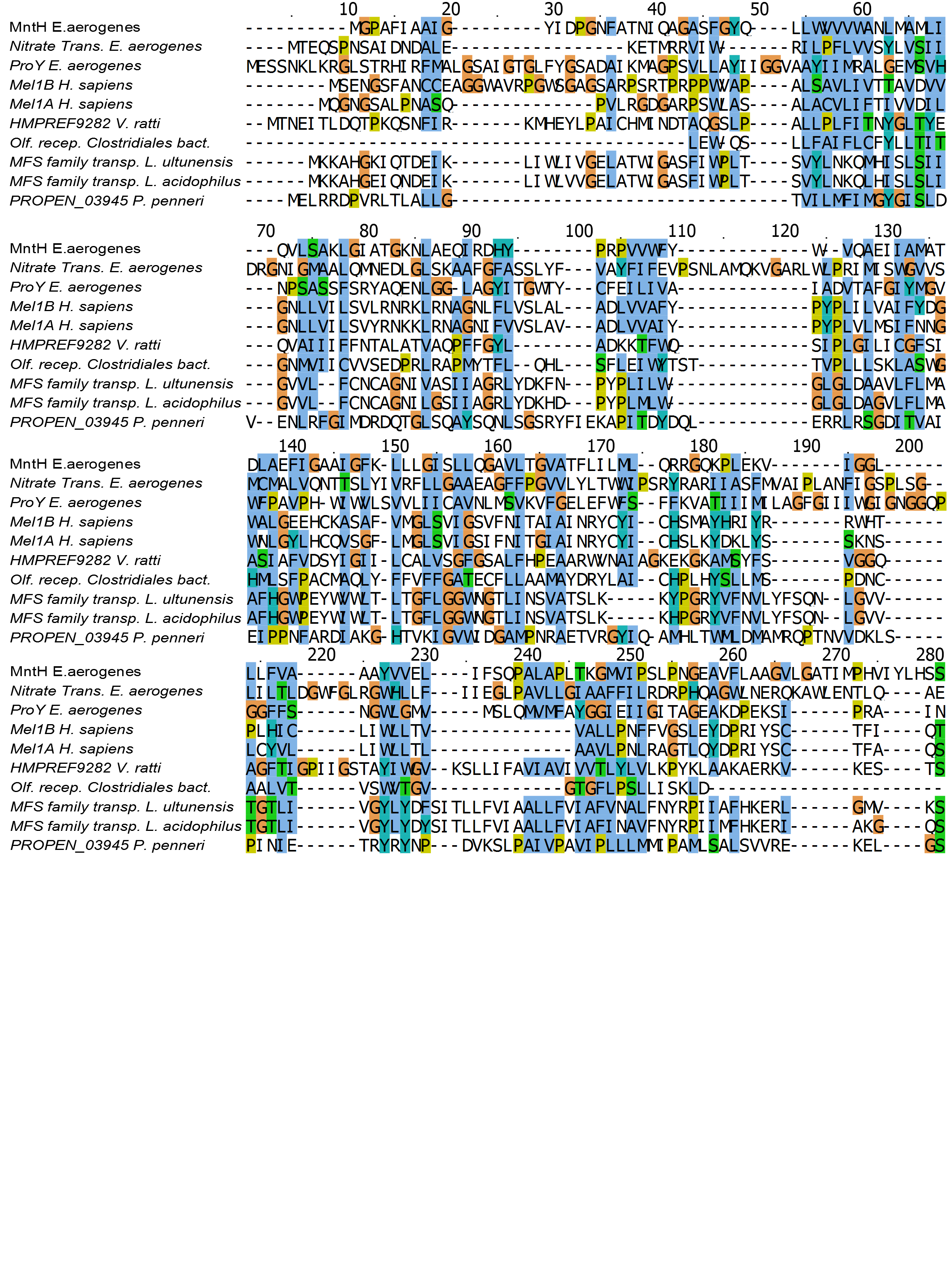

Supplement: S1 Fig — Alignments shown are a selection of positive BLAST hits (e-value < 0.001) aligned using MUSCLE that show several conserved residues and regions of high identity. (TIF) [file pone.0146643.s001.tif]

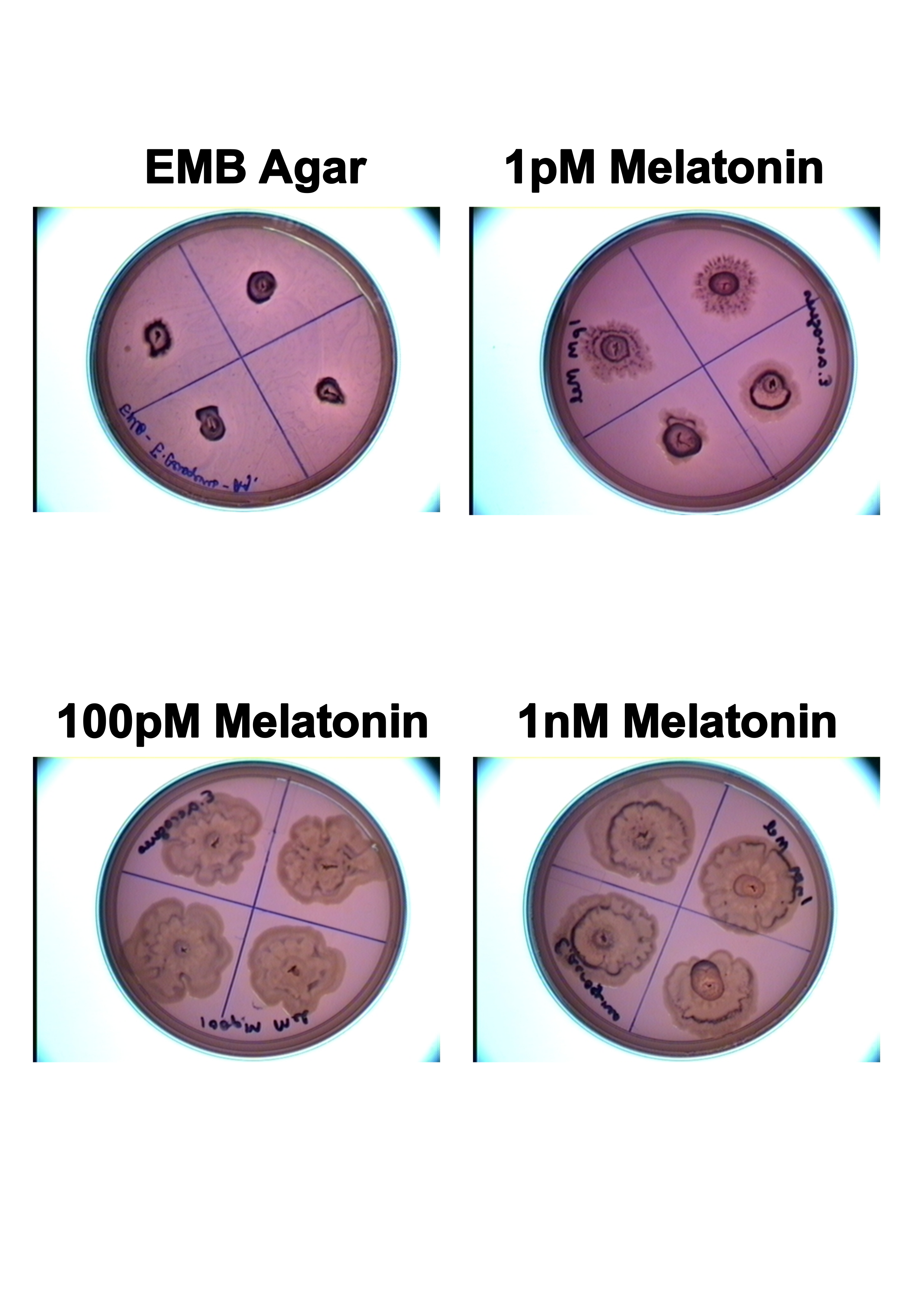

Supplement: S2 Fig — 100mm EMB agar plates were inoculated with 2ul of overnight cultures (n = 4/plate, replicated with 4 different starter cultures) and incubated for 48 hours. Rosette patterns of swarming increased with increasing concentrations of melatonin on the plates. (TIF) [file pone.0146643.s002.tif]

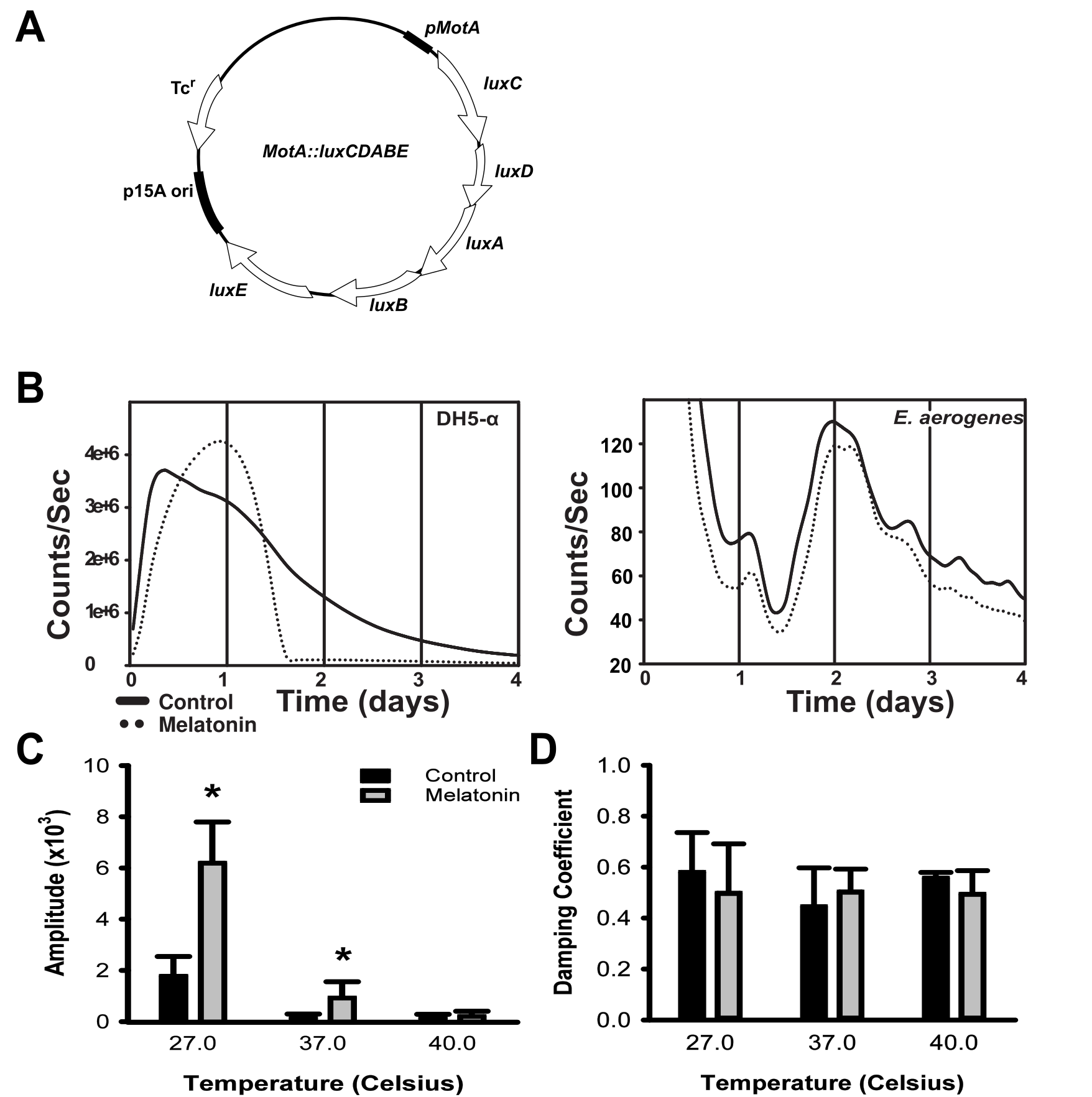

Supplement: S3 Fig — A Representative map of plasmid pRG19 showing MotA upstream of luxcdabe complex and tetracycline resistance. B) DH5-α cultures (left) are not rhythmic regardless of presence of melatonin, however, raw trace of E. aerogenes cultures (right) transformed with MotA::luxcdabe plasmid show rhythmic expression with damping over time both in the presence and absence of melatonin. C) Melatonin increased the average amplitude of cultures exhibiting circadian rhythms at 27°C and 37°C, but not 40°C, * = p value < 0.05 as tested by one-way ANOVA. D) Neither temperature nor melatonin affected the damping rate of the cultures exhibiting circadian rhythms. (TIF) [file pone.0146643.s003.tif]

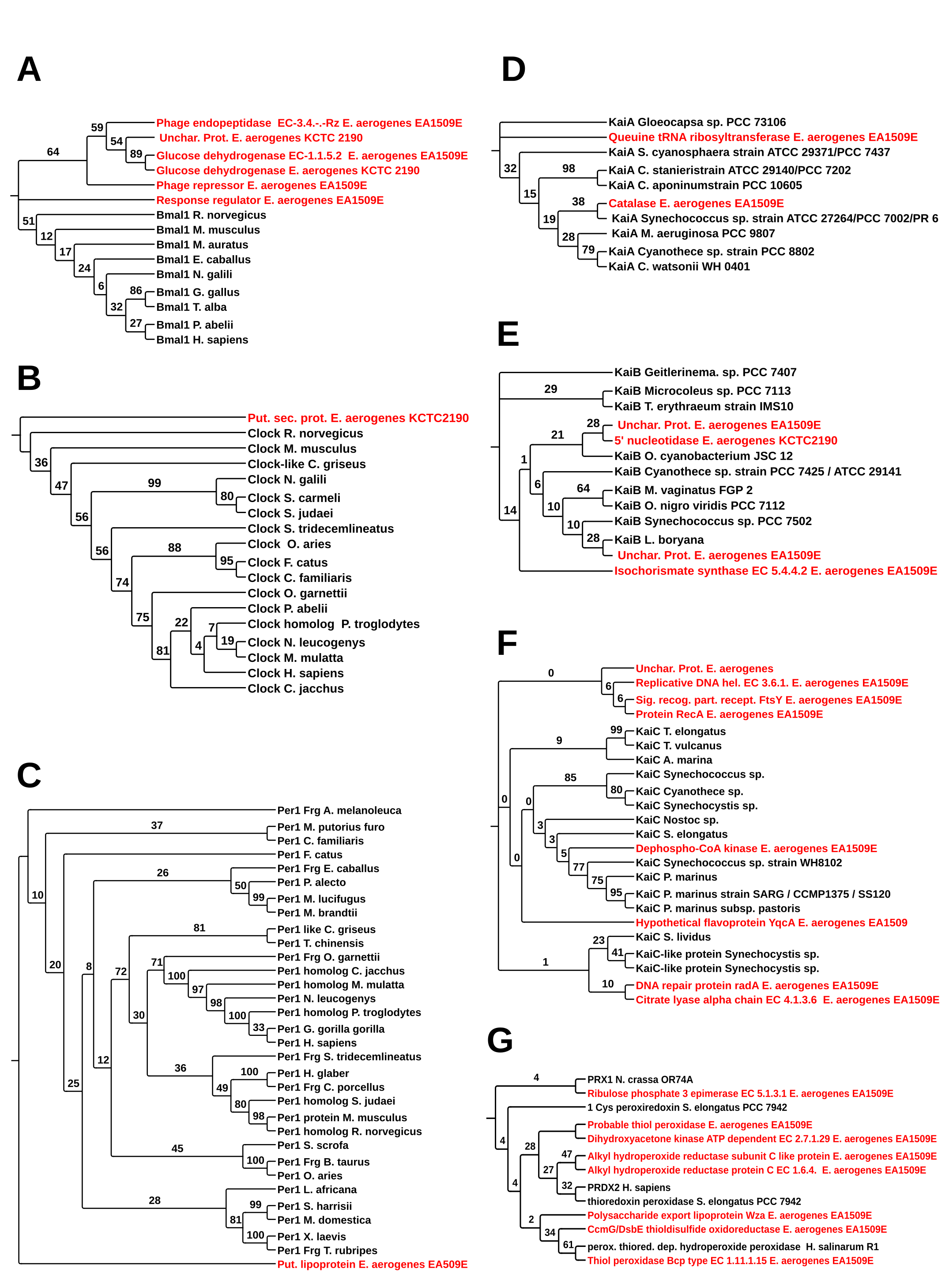

Supplement: S4 Fig — Bootstrapped trees (iterations shown between branches) show no homology among E. aerogenes proteins and vertebrate clock proteins BMAL1 (A), CLOCK (B), or PER1 (C). Similar analyses using Uniprot clusters of KAI A (D), KAI B (E), and KAI C (F) show potential homology with specific E. aerogenes proteins. G) E. aerogenes proteins share conservation with redox-related proteins across several taxa. (TIF) [file pone.0146643.s004.tif]

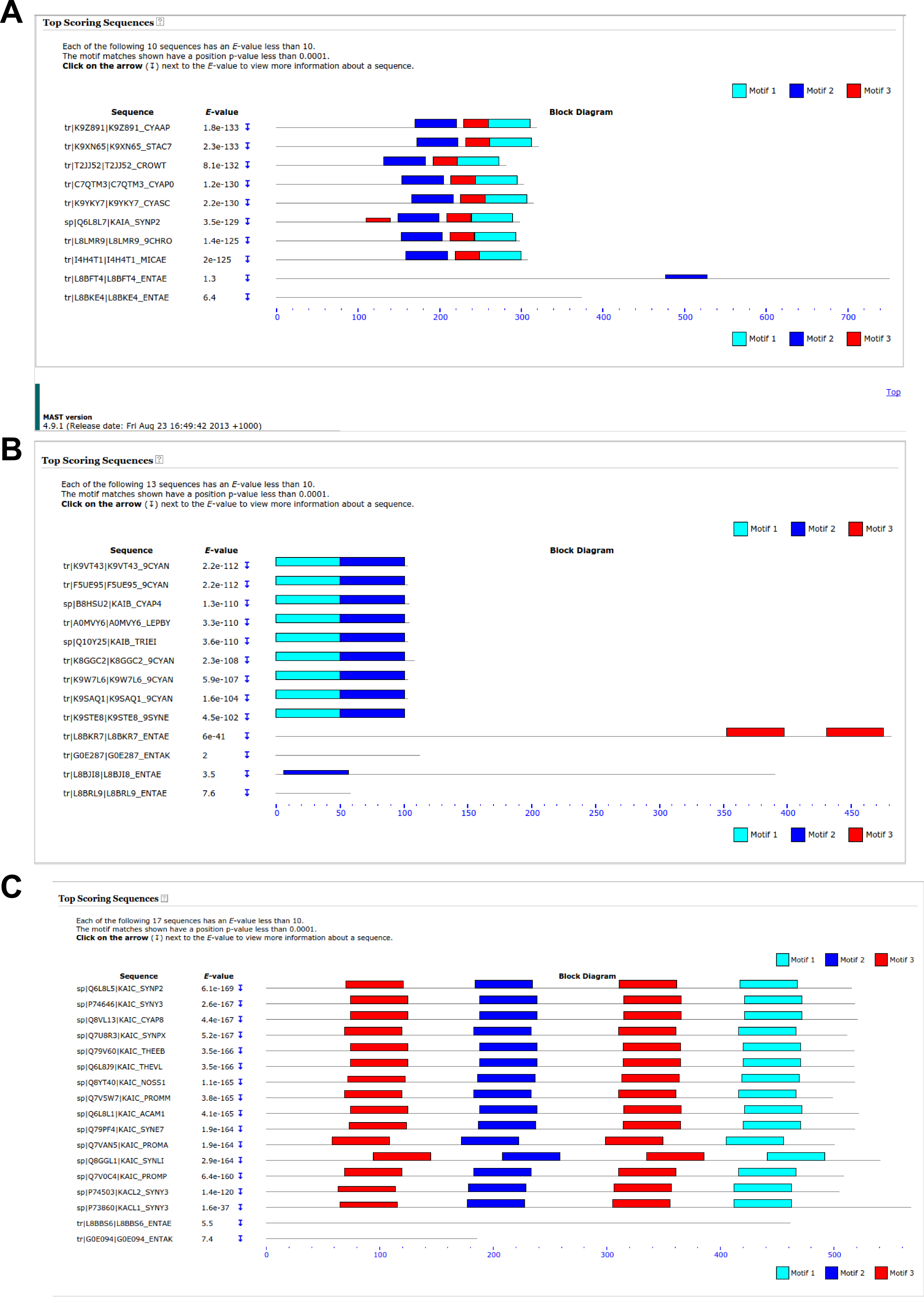

Supplement: S5 Fig — Proteins with sequence homology via PSI-BLAST share some motif-level sequences with A) KaiA and B) KaiB, but not C) KaiC. (TIF) [file pone.0146643.s005.tif]
